# Effects of tonifying kidney and strengthen bone therapy on non-dialysis patients with chronic kidney disease-mineral and bone disorder: A protocol for the systematic review and meta-analysis of randomized controlled trials

**DOI:** 10.1097/MD.0000000000024445

**Published:** 2021-02-12

**Authors:** Zijian Wu, Liang Li, Guiling Wu, Youqiong Xie, Jia Li, Rui Peng

**Affiliations:** a College of Acupuncture and Orthopedics; b Clinical College of Chinese Medicine, Hubei University of Chinese Medicine; c Wuhan Hospital of Traditional Chinese Medicine; d Hubei Provincial Collaborative Innovation Center of Preventive Treatment by Acupuncture and Moxibustion, Hubei University of Chinese Medicine, Wuhan, China.

**Keywords:** chronic kidney disease-mineral and bone disorder, protocol, randomized controlled trials, tonifying kidney and strengthen bone therapy, traditional Chinese medicine

## Abstract

**Background:**

Correction of calcium (Ca), phosphorus (P), and parathyroid hormone (PTH) disorders is the standard of treatment in non-dialysis patients with chronic kidney disease-mineral and bone disorder (CKD-MBD), but the side effects and adverse reactions brought by western medicine (WM) are still the main problems. More importantly, the lack of protection of kidney function in the treatment greatly affects the health of patients. Although traditional Chinese medicine (TCM), specifically tonifying kidney and strengthen bone (TKSB) therapy is wildly applied for patients with CKD-MBD in China, the evidence of TKSB therapy in the treatment of CKD-MBD is limited. Thus, we pretent to conduct this protocol to evaluate the efficacy and safety of TKSB therapy combined with WM for non-dialysis patients with CKD-MBD.

**Methods:**

A system research of randomized controlled trials (RCTs) of TKSB therapy for CKD-MBD will be conducted by 2 investigators from 7 electronic databases. Methodological quality evaluations will be performed by using the Cochrane collaboration tool and data analysis will be conducted by RevMan V5.3 software and STATA v15.0.

**Results:**

The results of this paper will be submitted to a peer-reviewed journal for publication.

**Conclusion:**

This research will determine the safety and efficacy of TKSB therapy in treating non-dialysis patients with CKD-MBD.

**INPLASY registration number:**

INPLASY2020120086

## 1 Introduction

Chronic kidney disease (CKD) is a major disease threatening human health. In the past 3 decades, the global incidence and prevalence of CKD have increased by 89% and 87%, respectively.^[1]^ In China, the prevalence of adult CKD is 13.4%.^[2]^ Such a high incidence and prevalence have brought a great burden to society. Chronic kidney disease-mineral and bone disorder (CKD-MBD) is one of the most common complications of chronic kidney disease.^[3]^ With the progression of CKD, the incidence of CKD-MBD is increasing. When it progresses to end-stage renal disease, almost all patients have renal bone disease (RBD).^[4]^ As early as CKD stage 3, due to the decline of renal function and the decrease of glomerular filtration rate, the renal phosphate excretion is impaired and the phosphate in the blood is retained. Such high phosphorus in the blood can directly inhibit the secretion of 1α-hydroxylase by the kidneys, leading to obstacles in the production of renal active vitamin D3.^[5]^ However, active vitamin D3 is an important component of intestinal absorption of calcium into the blood, so lack of it will cause hypocalcemia.^[6]^ The reduction of activated vitamin D3 can make bones have varying degrees of resistance to parathyroid hormone (PTH), which will increase the compensatory secretion of PTH in the blood. At this time, the increased PTH will mobilize a large amount of bone calcium into the blood to maintain the balance of Ca and P, but it will cause bone calcium loss in bone tissue, leading to osteoporosis, fractures, bone pain, and other bone diseases, which is the so-called CKD-MBD.^[7]^ These diseases have severely affected the quality of the patients’ life and shortened their survival time.

At present, the main clinical treatment methods for CKD-MBD are as follows^[8]^: reduce phosphorus intake and promote phosphorus clearance to control hyperphosphatemia; supplement calcium and active vitamin D3, adjust blood calcium to maintain a safe level and avoid the occurrence of hypercalcemia; prevention and treatment of secondary hyperparathyroidism; if the effect of drug therapy is not good, parathyroid surgery can be considered. These therapies listed above are effective in controlling calcium, phosphorus, and parathyroid hormone in the blood, but there are many adverse reactions. For example, the oral administration of aluminum hydroxide can easily cause the accumulation of aluminum in the body, and the improper grasp of the dosage and treatment course of calcium can easily cause hypercalcemia.^[9]^ Once in end-stage of CKD-MBD, parathyroidectomy for the treatment of tertiary hyperparathyroidism not only brings irreversible damage to the body, but also causes a heavy economic burden on the patient.^[10]^ More importantly, western medicine (WM) ignores the improvement of renal function, as well as the lack of early prevention of CKD-MBD. Patients often take treatment measures only when they have severe bone pain, bone deformity, and other clinical symptoms, but it is very difficult to reverse the course of the disease at this time. Therefore, it is essential to seek treatments which can both improve renal function and regulate Ca, P, and PTH levels.

Traditional Chinese medicine (TCM) is widely used in the prevention and treatment of kidney diseases and bone metabolism diseases. According to the theory of TCM, renal bone disease belongs to the category of “bone impotence” in TCM. Bone is closely related to kidney. Bone loss is the main characteristic of kidney deficiency. Kidney deficiency and bone atrophy are the basic pathological features of RBD. Therefore, the classic therapy of TCM for RBD is tonifying kidney and strengthen bone (Bushen Jiangu).^[11]^ The combination of TKSB therapy with WM can not only improve renal function, but also regulate the levels of Ca, P, and PTH. For example, Zheng et al^[12]^ found that Yishen Jiangzhuo Granule, an effective TCM for TKSB, combined with WM treatment, could significantly improve renal function by increasing CCr and decreasing SCr when compared with WM alone. Zhou^[13]^ showed that the combination of WM with TKSB therapy could improve the symptoms of renal function and reduce bone loss by increasing Ca levels in blood, reducing P and PTH levels in the blood, increasing bone density, and reducing ALP content. The therapeutic effect of combining treatment group was superior to the WM treatment group. All the above studies have shown that TKSB therapy pays more attention to improving renal function in the treatment of CKD-MBD, that is, it uses herbal drugs to match the kidney bias to improve the vitality of the kidney to help the body restore the normal metabolic function of the kidney, which is the essence of TCM in the treatment of CKD-MBD. It does not only focus on regulating the abnormal trace elements in the blood as doing like this will ignore the analysis of pathogeny, leading to the gradual decline of renal function and ultimately resulting in an irreversible situation of dialysis treatment. Inspired by these findings, we wonder if the treatment is focused on improving renal function, perhaps the trace elements in the blood will return to normal, which is what TCM is good at and pursues. At present, a large number of literature have reported the clinical efficacy and drug safety evaluation of TKSB therapy in the treatment of CKD-MBD, including dialysis patients and non-dialysis patients. Since metabolism is not carried out through the kidney in dialysis patients, the effect of TCM on renal function improvement cannot be reflected in the body of dialysis patients. Based on this point, we believe that the therapeutic effect of TKSB therapy on non-dialysis patients is more significant. However, there has been no meta-analysis to evaluate the clinical efficacy of TKSB therapy in the treatment of CKD-MBD in non-dialysis patients. Consequently, our study is to lay a foundation for promoting the clinical application of TKSB in the treatment of CKD-MBD in non-dialysis patients.

## 2 Methods

This protocol is registered in INPLASY (INPLASY2020120086). This protocol was performed in accordance with the preferred reporting items for systematic reviews and meta-analysis protocol. Ethical approval is unnecessary because this is a literature-based study.

### 2.1 Data sources and search strategy

We will comprehensively search the following 7 databases: PubMed, EMBASE, Cochrane Library, Wanfang database, Chinese National Knowledge Infrastructure (CNKI), China Biological Medicine (CBM), and VIP Journals Database. The basic terms were performed as follows: (bushen OR yishen OR tonifyingkidney OR bugu OR jiangu OR zhuanggu OR strengthen bone) AND (Renal Osteodystrophy OR renal bone disease OR renal bone disorder OR kidney bone disease). Ambiguous literature will be manually searched to avoid missing eligible trials. Ongoing registered clinical trials will be searched on the websites of the Chinese clinical trial registry (http://www.chictr.org.cn/), and international clinical trial registry (http://clinicaltrials.gov/). We will contact the original investigators for more complete details of the study to solve questions about eligibility if necessary.

### 2.2 Inclusion criteria and study selection

#### 2.2.1 Participants

Patients with CKD-MBD who do not receive renal dialysis treatment will be included. Patients who did not meet the diagnostic criteria or received dialysis treatment in the treatment group or control group will be excluded.

#### 2.2.2 Interventions

The treatment must include one type of TKSB therapy (e.g., bushen, yishen, tonifyingkidney, bugu, jiangu, zhuanggu, strengthen bone) combined with one type of WM (e.g., calcium carbonate tablets, calcitriol).

#### 2.2.3 Comparisons

The control group should receive WM treatment alone. If the control group contains other TCM therapy or dialysis treatment, it will be excluded.

#### 2.2.4 Outcomes

Included studies should contain at least 4 of the following evaluated outcomes: clinical effective rate; serum Ca level; serum P level; parathyroid hormone (PTH); blood urea nitrogen (BUN) level; serum creatinine (SCr) level; adverse events from TKSB therapy or WM.

#### 2.2.5 Study design

**Figure 1:**
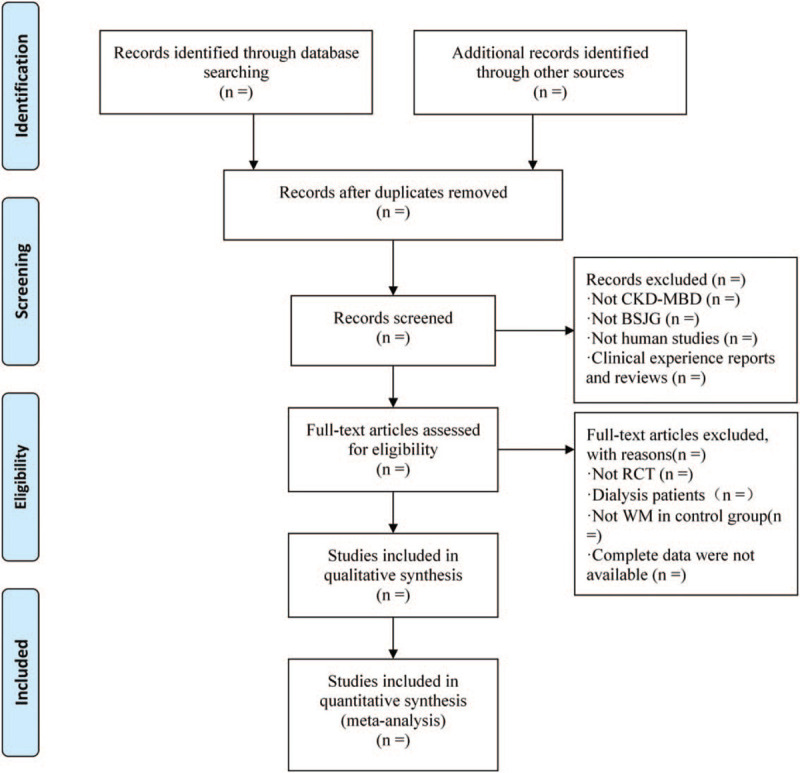
Flow diagram of study selection.

Randomized controlled trial and quasi- randomized controlled trials (RCT) including combination therapy of TKSB will be included. Articles will be excluded if they are case reports, letters, editorials, and nonhuman studies. The flow diagram of the study selection is shown in Fig. 1.

### 2.3 Data extraction

For eligible studies, the following data will be independently extracted by 2 authors (GLW and LL) according to predefined criteria: first author, publication year, region, sample size, detail of intervention, treatment courses, and outcome parameters. For any disagreement between the 2 authors, it would be settled through discussion with a third author (YQX). The reasons for exclusion were recorded. The data were extracted from the included RCTs to a predefined Excel table (Microsoft Corp., Redmond, WA) and cross-checked by the 2 reviewers (GLW, JL). In the event of missing data, we will attempt to contact the corresponding authors for details.

### 2.4 Assessment of risk of bias and quality assessment in included studies

Two authors (GLW, YQX) independently assessed the methodological quality of each trial according to the standards advised by the Cochrane Handbook.^[14]^ Disagreements, if any, were resolved by discussion and reached consensus through a third reviewer (JL). The assessed items included 6 domains: random sequence generation; allocation concealment; blinding method; integrity of data; selective reporting; other bias. Selective reporting bias was judged according to the published protocols for the registered clinical trials that were contained on the Chinese clinical trial registry (http://www.chictr.org) and international clinical trial registry of the US National Institutes of Health (http://clinicaltrials.gov) websites. We compared the outcome measures between the study protocol and the final published trial.

### 2.5 Data analysis

Review Manager 5.3 (The Nordic Cochrane Centre, Copenhagen, Denmark), compiled by the Cochrane Collaboration, will be employed to pool and analyze data. Dichotomous variables will summarize as risk ratios (RR) with 95% confidence intervals (CI), while continuous variables will summarize as mean difference (MD) with 95% CI. Heterogeneity will be examined by the *I*^2^ test. The result of *I*^2^ test above 50% will be considered to indicate significant heterogeneity, and the meta-regression model will be applied to find out the sources of the heterogeneity. Additionally, if necessary, a sensitivity analysis will be carried out to evaluate the stability and reliability of the results by removing individual studies at a time.

### 2.6 Assessment of publication bias

The funnel plot to evaluate the potential publication bias will be used when there are >10 studies included in the meta-analysis. And quantitative analysis of publication bias will measured by the method of Egger test or Begg test.

## 3 Discussion

RBD is one of the most common and serious complications of CKD. Metabolic disorders such as Ca, P, and PTH in vivo caused by chronic renal impairment will lead to cardiovascular diseases and fractures, posing a great threat to human life and health.^[15]^ However, the optimal strategy for the treatment of CKD-MBD remains controversial. For example, Gross et al^[16]^ believe that there is still no convincing RCT evidence to confirm whether reducing the level of P in the blood can improve the clinical prognosis and reduce the mortality of patients with CKD-MBD. Block et al^[17]^ believed that the use of calcium-phosphorus binder or non-calcium-phosphorus binder in patients with CKD stage 3–4 could accelerate the rate of vascular calcification, and it was difficult to obtain satisfactory clinical efficacy. In this context, the current treatment of CKD-MBD only focuses on regulating the metabolic levels of Ca, P, and PTH in the blood, while ignoring the improvement of renal function and considering its adverse effects. At present, TCM is widely used to improve the symptoms and signs of patients with CKD-MBD, and it is especially good at improving patients’ physical conditions and improving their quality of life. According to the theory of TCM, the incidence of CKD-MBD is usually in the kidney and bone, and its pathogenesis is closely related to kidney deficiency and bone impotence, which mostly belong to deficiency syndrome. Thus, TKSB therapy is the main method for TCM treatment of CKD-MBD. In recent years, increasingly RCTs have been reported on the treatment of CKD-MBD with TKSB therapy, which provides an opportunity for the objective and comprehensive evaluation of this method. In order to clarify the efficacy and safety of this treatment strategy in a scientific and reasonable way so as to promote its clinical application, we pretent to conduct a meta-analysis in the future. Through the forthcoming meta-analysis, we will conduct a statistical analysis of the herbs used at a high frequency to provide a better therapy.

As the systematic review is based on the secondary research of published literature, there are undeniable methodological defects. In addition, the quality of the included studies determines the quality level and reliability of the final results. We will begin to conduct the review when the necessary trials are met, and all operating procedures will be performed in accordance of Cochrane Handbook to ensure that the provided information is helpful for clinicians and patients.

## Acknowledgments

The authors would like to thank Professor Youping Li, Director of Cochrane Center in China; Professor Rui Peng, Director of College of Acupuncture and Orthopedics of Hubei University of Chinese Medicine in China; Professor Jia Li, Director of Hubei Provincial Collaborative Innovation Center of Preventive Treatment by Acupuncture and Moxibustion in China for their training on Cochrane system evaluation and knowledge of statistical analysis.

## Author contributions

**Data curation:** Liang Li.

**Formal analysis:** Guiling Wu.

**Methodology:** Jia Li.

**Software:** Youqiong Xie.

**Supervision:** Rui Peng.

**Writing – original draft:** Zi Jian Wu.

## Abbreviations

Abbreviations: BUN = blood urea nitrogen, Ca = calcium, CI = confidence interval, CKD-MBD = chronic kidney disease-mineral and bone disorder, MD = mean difference, P = phosphorus, PTH = parathyroid hormone, RBD = renal bone disease, RCT = randomized controlled trials, RR = risk ratio, SCr = serum creatinine, TCM = traditional Chinese medicine, TKSB = tonifying kidney and strengthen bone, WM = western medicine.
